# *Elizabethkingia* in the MENA region (2000–2024): a review of distribution, antimicrobial resistance, and clinical features

**DOI:** 10.1186/s12879-025-11420-5

**Published:** 2025-08-21

**Authors:** Abdallah Alhaj Sulaiman, Mouayad Zuheir Bakleh, Reem Ali, Mustapha Aouida, Dindial Ramotar

**Affiliations:** 1 Division of Biological and Biomedical Sciences, College of Health and Life Sciences, Hamad Bin Khalifa University, Education City, Qatar Foundation, P.O. Box 34110, Doha, Qatar; 2 Division of Genomics and Precision Medicine, College of Health and Life Sciences, Hamad Bin Khalifa University, Education City, Qatar Foundation, P.O. Box 34110, Doha, Qatar; 3 Translational Oncology Research Center, Qatar Biomedical Research Institute (QBRI), Hamad Bin Khalifa University (HBKU), Qatar Foundation (QF), P.O. Box 34110, Doha, Qatar

**Keywords:** *Elizabethkingia*, Antimicrobial resistance, MENA region

## Abstract

*Elizabethkingia* is a family of bacteria present in the environment causing infections that have emerged as a significant clinical concern in the Middle East and North Africa region. Despite sporadic reports and case studies, the epidemiology of Elizabethkingia—encompassing its prevalence, antimicrobial resistance profiles, and clinical outcomes—remains largely anecdotal and fragmented. In this review, we systematically screened and analyzed the published literature to provide a comprehensive assessment of the distribution, antibiotic resistance characteristics, and clinical features of *Elizabethkingia* infections in different samples across the region. Our analysis consolidates existing evidence to highlight the true burden of *Elizabethkingia* and the emergence of multidrug-resistant strains. By identifying current gaps in data and standardization, this review aims to inform and engage clinicians, microbiologists, epidemiologists, and public health stakeholders in developing coordinated strategies to monitor, prevent, and manage these infections within a One Health framework.

**Supplementary Information:**

The online version contains supplementary material available at 10.1186/s12879-025-11420-5.

## Introduction

*Elizabethkingia meningoseptica* is a Gram-negative, rod-shaped bacterium that was originally classified as *Flavobacterium meningosepticum*. It was first isolated in 1949 from the cerebrospinal fluid, blood, and throat swabs. Elizabeth O. King subsequently described the bacterium in March 1959 [1]. In a seminal study published in 1994, Vandamme and colleagues re-examined the genotypic, chemotaxonomic, and phenotypic characteristics of the *flavobacteria*. Their analysis revealed that many species traditionally assigned to *Flavobacterium*, including *F. meningosepticum*, did not belong together. As a result, they proposed the new genus *Chryseobacterium* to accommodate several of these misclassified organisms—with *Chryseobacterium gleum* designated as the species type [2]. Building on these findings, further studies employing polyphasic approaches—combining 16S rRNA gene sequencing, fatty acid analysis, and DNA–DNA hybridization—demonstrated that the lineage containing *F. meningosepticum* was distinct from other *Chryseobacterium* species. In 2005, Kim and colleagues used these methods to show that both *Chryseobacterium meningosepticum* and *Chryseobacterium miricola* formulate a separate branch within the family *Flavobacteriaceae* [3]. They proposed transferring these species to a new genus, Elizabethkingia, in honor of Elizabeth O. King’s pioneering work [3]. As a result, the bacterium formerly known as *Chryseobacterium meningosepticum* was renamed *Elizabethkingia meningoseptica*. This re-classification reflects a more accurate understanding of the evolutionary relationships within this group and has important implications for diagnosis, treatment, and epidemiological tracking of infections caused by these organisms.

Following the re-classification of *Chryseobacterium meningosepticum* to *Elizabethkingia meningoseptica* in 2005, researchers began identifying additional species within this newly defined genus. One of the initially described species was *Elizabethkingia anophelis*, which was isolated from the midgut of the mosquito *Anopheles gambiae* in 2011 [4]. Using a polyphasic approach that included 16S rRNA gene sequencing, fatty acid analysis, and DNA–DNA hybridization, Kämpfer and colleagues demonstrated that strain R26^T, though closely related to *E. meningoseptica* and *E. miricola*, was distinct enough to warrant its classification as a new species—*Elizabethkingia anophelis* [4]. Subsequent genomic studies further expanded the genus. In 2017, Nicholson et al. revisited the taxonomy of *Elizabethkingia* using whole-genome sequencing, optical mapping, and MALDI–TOF MS [5]. Their work led to the proposal of three additional species: *Elizabethkingia bruuniana*, *Elizabethkingia ursingii*, and *Elizabethkingia occulta*. These discoveries aided in the clarification of the genetic diversity within the genus and improved species-level differentiation for clinical and epidemiological purposes [5].

Taking into account that understanding the specific geographical attributes of antimicrobial resistance and bacterial infections is essential for tailoring public health responses, it is expected that global trends may not fully capture the nuances present in individual regions [6]. Also, despite the advances in our understanding of the taxonomy of *Elizabethkingia*—relatively little is known about its distribution and clinical impact in the Middle East and North Africa (MENA) region. Reports of *Elizabethkingia* infections, including outbreaks and sporadic cases, have emerged from various parts of the world. However, studies focusing on the MENA region remain scarce. This review aims to consolidate the available evidence on *Elizabethkingia* infections in the MENA region, identify common clinical presentations and antimicrobial resistance patterns, and outline the public health implications for the region. To this end, we systematically reviewed the literature from relevant databases and regional reports to map out the current state of knowledge. This review provides insight into the prevalence and characteristics of *Elizabethkingia* infections in the MENA region and also highlights critical gaps that need to be addressed by future research.

## Methodology

The literature search for this review was performed on October 9, 2024, using a set of databases accessed on the same day: Cochrane (https://www.cochrane.org), PubMed (https://pubmed.ncbi.nlm.nih.gov), Scopus (https://www.scopus.com), Web of Science (https://webofscience.com), and Arab World Research Source (https://www.ebsco.com). To capture a comprehensive set of terms, Medical Subject Headings (MeSH) were applied to identify synonyms for both bacterial naming and regions. A detailed description of the search strategy and the specific terms employed is provided in Supplementary File S1, sheet 1.

For this study, articles were considered for inclusion if they met the following criteria: research conducted in countries within the MENA region; publications available in English or Arabic or provided with a translation; and original research articles such as case reports, case series, cross-sectional studies, cohort studies, randomized controlled trials, and letters to editors. Studies were required to report on *Elizabethkingia* isolated from any source, including clinical specimens, environmental samples, or other materials, and to include data concerning prevalence and/or characteristics (such as antimicrobial susceptibility, genetic profiles, and clinical outcomes) of the organism. Additionally, studies published within the designated timeframe (from 2000 to 2024) were considered eligible. Conversely, studies were excluded if they were conducted outside the MENA region, published in languages other than English or Arabic without available translation, or were review articles, meta-analyses, editorials, commentaries, or conference abstracts lacking full-text availability. Publications that did not provide specific data on *Elizabethkingia*, focused on unrelated organisms or were duplicate reports were also excluded, likewise for studies with inaccessible full texts or those published before 2000.

Articles retrieved from the search were first imported into EndNote 20.4 to eliminate duplicates and then exported into an Excel spreadsheet containing essential details—such as Region, Causative Agent, Resistance Profile, Sensitivity, Disease, Source, Method of Identification, Treatment Outcome, Virulence Factor, AMR Genes, Plasmids, and Symptoms —for further evaluation (Supplementary File S1). The screening process was executed in two stages by one author and subsequently verified independently. Initially, titles and abstracts were reviewed to discard studies that did not align with the predefined criteria (Supplementary File S1, sheet 2). Thereafter, full texts of the remaining articles were assessed to determine their eligibility for data extraction and analysis (Supplementary File S1, sheet 3). Data extraction followed the PICOS framework: the Population encompassed any sample type related to *Elizabethkingia* within the MENA region; the Intervention involved the isolation and identification of *Elizabethkingia* from various sources; Comparison, when applicable, included differences in strains, sources, countries, or periods; and Outcomes considered prevalence rates, antimicrobial susceptibility, clinical outcomes, and epidemiological data. The study design incorporated any primary research, including case reports, cross-sectional studies, cohort studies, trials, and relevant letters to editors. All extracted data were manually compiled and independently reviewed to ensure consistency and accuracy, with additional cross-referencing conducted using supplementary materials where necessary.

We utilized Python with Pandas and Matplotlib softwares to generate a stacked bar chart that displays the number of studies published per year by region. The raw data were defined as a list of dictionaries and loaded into a Pandas DataFrame, from which records with missing region data were removed. The data were then grouped by “Year” and “Region” to count occurrences and pivoted so that each region formed a separate column. Finally, a stacked bar chart was plotted using Matplotlib and saved at high resolution for publication purposes.

The same packages were used along with Seaborn for generating heatmaps. Raw data, comprising region-specific antibiotic profiles for resistance and sensitivity, were imported into a pandas DataFrame and preprocessed by splitting comma-separated antibiotic strings into individual tokens. Each antibiotic was then classified into its corresponding family (e.g., Penicillins, Cephalosporins, Carbapenems, Aminoglycosides, Fluoroquinolones, etc.) using a custom dictionary mapping and a function that normalized names to lowercase and matched substrings; entries that did not match any category were labeled as “Unknown” and excluded from further analysis. The data were transformed into a long-format DataFrame, with each row representing a single antibiotic occurrence along with the associated region and category (Resistant or Sensitive). Counts were aggregated by grouping of region, category, and antibiotic family and then normalized to percentages within each region–category combination. Finally, heatmaps were generated using seaborn with a reversed grayscale colormap (white representing 0% and black higher percentages) and custom annotations displaying percentage values only when nonzero. Separate heatmaps were produced for Qatar, Saudi Arabia, and Turkey, as well as for all other regions. A nested donut chart was created by overlaying two concentric pie charts using Python’s matplotlib library. The data were analyzed manually and fed to the code to generate the figure.

## Results

### Study selection and publication trends

**Fig. 1:**
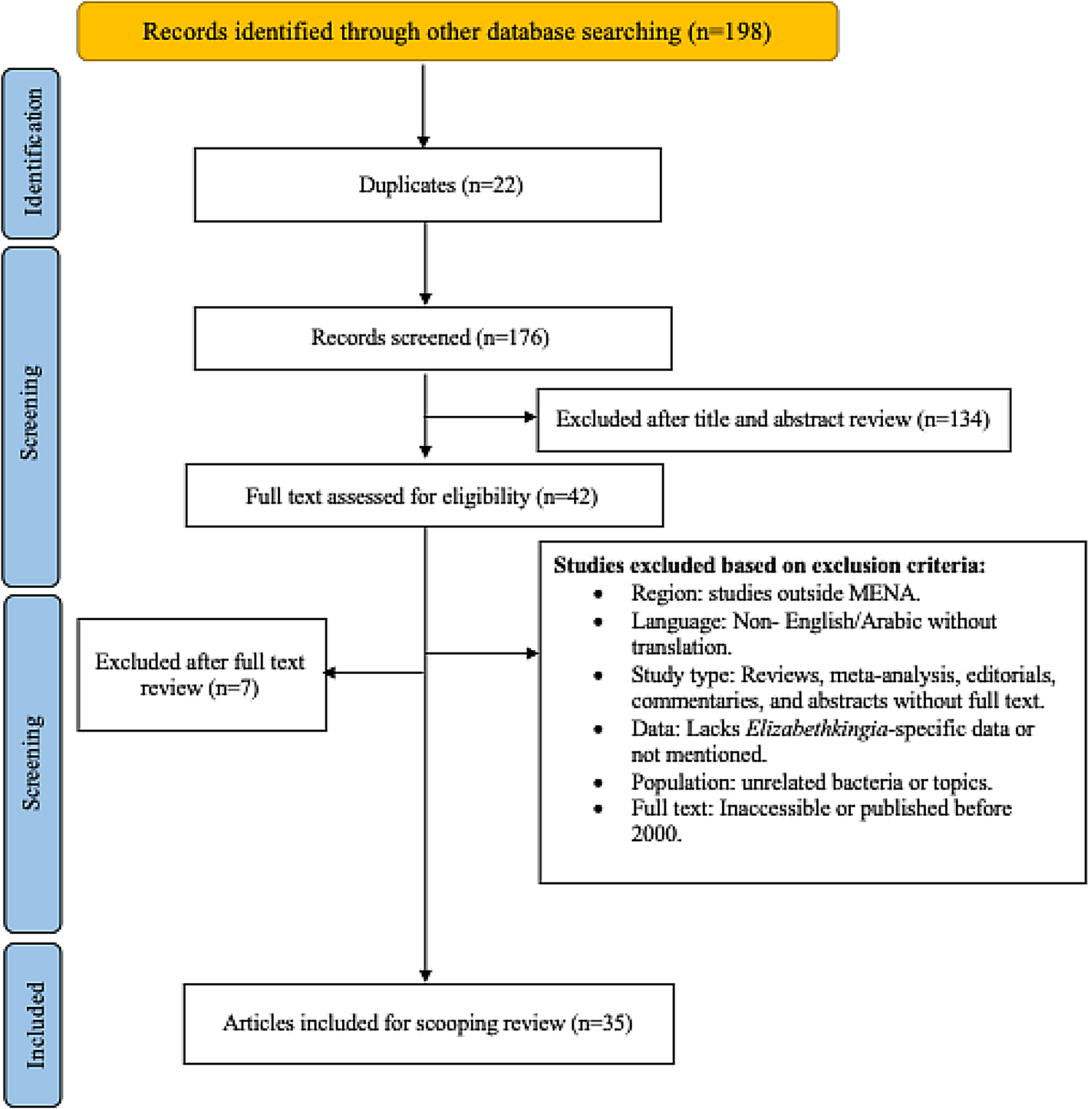
PRISMA flow diagram illustrating the step-by-step process of study selection. The figure depicts the identification of records from database searches, the removal of duplicates, and the subsequent screening, eligibility, and inclusion stages. It also specifies how many articles were excluded and how many were ultimately included in the final review [7]

**Fig. 2:**
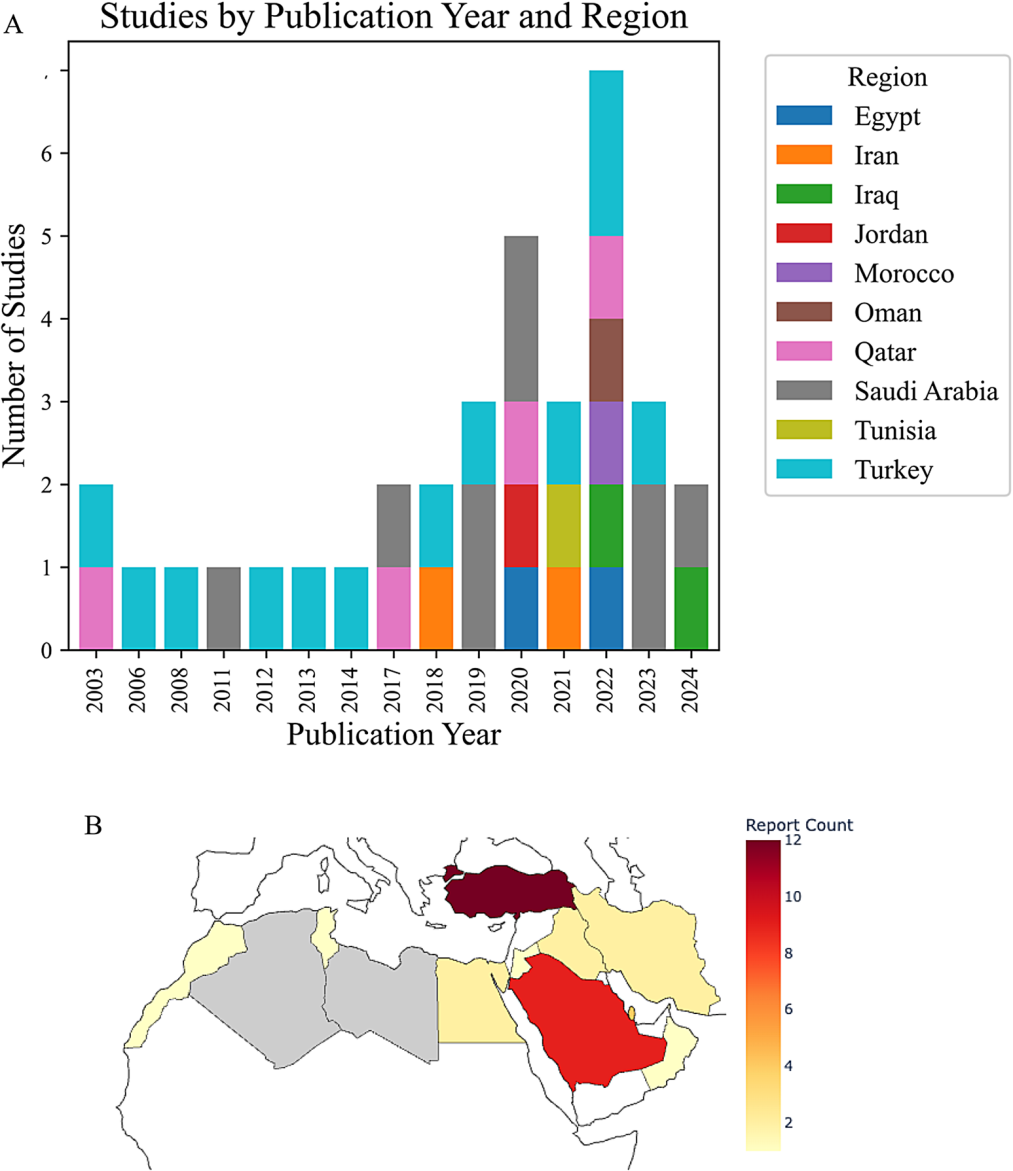
Distribution of *Elizabethkingia* Studies by Country in the MENA Region. **A** Number of *Elizabethkingia*-related studies published each year (2000–2024), grouped by country of origin in the MENA region. Each bar corresponds to the total number of studies in a given year, with different colors representing individual countries. **B** Choropleth map of the MENA region, with countries shaded on a YlOrRd scale according to their publication counts; grey indicates countries without any publications

A total of 198 records were identified through database searches. After removing 22 duplicate records, 176 unique articles remained for screening. Of these, 134 were excluded following a review of titles and abstracts, leaving 42 articles for full-text assessment. Subsequently, 7 articles were excluded for reasons including region (studies outside the MENA area), language restrictions, inappropriate study design, lack of *Elizabethkingia*-specific data, and inaccessible full texts or publication dates before 2000. Ultimately, 35 articles satisfied all eligibility criteria and were included in the final scoping review (Fig. 1). Following the final inclusion of 35 articles, the distribution of these studies by publication year and country is illustrated in (Fig. 2A, B). The earliest reports appeared in 2003, originating from both Qatar and Turkey. Sporadic contributions from Turkey continued in 2006, 2008, 2012, 2013, and 2014, while Saudi Arabia first emerged in the literature in 2011. Multiple countries began reporting *Elizabethkingia* research more frequently starting in 2017, including Qatar and Saudi Arabia again, and new additions such as Iran (2018) and Tunisia (2021). By 2022, a broader array of countries—Egypt, Iraq, Morocco, Oman, and Turkey—contributed at least one publication each, representing the most diverse single-year spread in the dataset. The trend persisted into 2023 and 2024 with continued outputs from Saudi Arabia, Iraq, and Turkey. Overall, Turkey reported the largest number of studies (12), followed by Saudi Arabia (9) and Qatar (4). Other countries in the region, such as Egypt, Iran, Iraq, Jordan, Tunisia, Morocco, and Oman, contributed fewer publications, while several MENA countries, such as Kuwait, Yemen, and Bahrain had no reports at all. This uneven distribution highlights both a growing regional interest in studying *Elizabethkingia* infections and significant data gaps in other parts of the MENA region, emphasizing the need for enhanced surveillance and research efforts.

**Table 1:** Reported *Elizabethkingia*-associated infections across MENA Countries

Country	Causative agent	Underlying conditions and risk factors	Source	Treatment outcome	Signs and symptoms	References
Egypt	*Elizabethkingia meningoseptica*	Not mentioned	Well Water	Not mentioned	Not mentioned	[8, 9]
	*Elizabethkingia miricola*	Not mentioned	Wastewater	Not mentioned	Not mentioned	
Iran	*Elizabethkingia meningoseptica*	Lymphangioleiomyomatosis	2-Year-old child	Recovery	Fever, Tachypnea	[10, 11]
	*Elizabethkingia anophelis*	Not mentioned	*Microcerotermes diversus* Silvestri (Termitidae)	Not mentioned	Not mentioned	
Iraq	*Chryseobacterium meningosepticum*	Not mentioned	Oil-Contaminated and Uncontaminated Sites	Not mentioned	Not mentioned	[12, 13]
	*Elizabethkingia meningoseptica*	Not mentioned	Cage-Cultured Common Carp	Not mentioned	Not mentioned	
Jordan	*Elizabethkingia meningosepticum*	Not mentioned	Treated Wastewater	Not mentioned	Not mentioned	[14]
Morocco	*Chryseobacterium meningosepticum*	Not mentioned	Hemodialysis Water Treatment System	Not mentioned	Not mentioned	[15]
Oman	*Elizabethkingia meningoseptica*	Neonatal Bacterial Sepsis	Neonate	Recovery	Respiratory Distress, Convulsions, Apnea	[16]
					Poor Feeding, Fever, Vomiting	
Qatar	*Flavibacterium meningococcus*	Meningitis	Neonate (< 30 days old)	Not mentioned	Hypoactivity, Drowsiness, Irritability, Vomiting, Fever	[17–20]
	*Chryseobacterium meningosepticum*	Could not be extracted	Infant (< 1 year old)	Death	Not mentioned	
	*Elizabethkingia meningoseptica*	Hemophagocytic Lymphohistiocytosis, Hemorrhagic Colitis	6-Year-old child	Death	Not mentioned	
	*Elizabethkingia meningoseptica*	Chronic Obstructive Pulmonary Disease, COVID-19	80-Year-old patient	Death	Severe Respiratory Distress, Hypoxia, Lung Opacities	
		Severe COVID-19 Pneumonia	49-Year-old patient		Fever, Cough, Severe Respiratory Distress	
		Severe COVID-19 Pneumonia	56-Year-old patient		Fever, Cough, Respiratory Distress, Sepsis, Arrhythmias, Cardiac Arrest, Seizures, Coma	
Saudi Arabia	*Chryseobacterium meningosepticum*	Not mentioned	Infant	Recovery	Lethargy	[21–29]
	*Elizabethkingia meningoseptica*	Biliary Drain Infection Following Percutaneous Transhepatic Cholangiography	55-Year-old female patient	Recovery	Right Upper Quadrant Abdominal Pain	
	*Chryseobacterium meningosepticum*	Sickle-Cell Anemia, Abdominal Surgery, Cardiac Surgery, Tracheoesophageal Surgery, Quadriplegia, Chest Fibrosis, Congenital Central Hypoventilation Syndrome, Bowel Ischemia, Parkinsonism, Heart Failure, Bacteremia	Patients aged 12 days to 102 years	10 Recovered, 2 Died	Not mentioned	
	*Elizabethkingia meningoseptica*	Hydatid Disease, Liver Cirrhosis, Malignancy, Pancreatitis	Patients aged 28 to 87 years	Not mentioned	Not mentioned	
	*Elizabethkingia meningoseptica*	Septic Shock, Bacteremia	22 patients (12 pediatric, 10 adult)	Not mentioned	Not mentioned	
	*Elizabethkingia meningoseptica*	Meningitis, Sepsis	Neonate	Recovery	Tachypnea, Fever, Decreased Consciousness, Anemia, Metabolic Acidosis	
	*Elizabethkingia meningoseptica*	Microbial Keratitis	73-year-old patient	Recovered with Faint Superficial Scar at Infiltrate Site	Loss of Vision	
	*Elizabethkingia meningoseptica*	Not mentioned	2 patients (1 pediatric, 1 elderly)	Not mentioned	Not mentioned	
	*Elizabethkingia meningoseptica*	Respiratory Disease (40.9%), Cardiovascular Disease (39.4%)	66 Patients (35 pediatric, 31 adult)	43 Recovered, 23 Died	Fever, Tachycardia, Hypotension, Respiratory Distress, Lethargy, Gastrointestinal Symptoms	
Tunisia	*Elizabethkingia meningoseptica*	Systemic Hypertension, Chronic Kidney Disease Stage 5	65-Year-old patient	Recovery	Persistent Abdominal Pain, Cloudy Peritoneal Dialysis Effluent	[30]
Turkey	*Chryseobacterium meningosepticum*	Stage 3 Hypoxic-Ischemic Encephalopathy, Sepsis	Four neonates (< 40 weeks gestational age)	3 Recovered, 1 Died	Sepsis, Respiratory Failure, Hypoxic-Ischemic Encephalopathy	[31–42]
	*Chryseobacterium meningosepticum*	Thalassemia Major, Meningitis, Sepsis	17-year-old male patient	Recovery	Fever, Headache, Altered Consciousness, Toxic Appearance, Meningeal Signs	
	*Chryseobacterium meningosepticum*	Meningitis, Gastroenteritis, Sepsis, Respiratory Distress, Postoperative Cellulitis/Fasciitis, Bacteremia, Pneumonia, Pneumothorax	Patients aged 4 days to 23 months, hand culture, formula, electric button, computer keyboard, telephone receiver, door knob, Ambu Bag	9 Recovered, 4 Died	Not mentioned	
	*Elizabethkingia meningoseptica*	Not mentioned	Neonate	Recovery	Abdominal Distention, Reduced Activity	
	*Chryseobacterium meningosepticum*	Septic Shock, Intractable Metabolic Acidosis, Multiple Organ Dysfunction Syndrome	Neonate	Death	Septic Shock, Metabolic Acidosis, Disseminated Intravascular Coagulation	
	*Elizabethkingia meningoseptica*	Cystic Fibrosis, Dilated Cardiomyopathy	Patients aged 7 to 15 months	Not mentioned	Not mentioned	
	*Elizabethkingia meningoseptica*	Persistent Patent Ductus Arteriosus, Respiratory Distress Syndrome, Late-Onset Nosocomial Neonatal Sepsis	26-Day-old patient, two neonates (< 24 h old)	Recovery	Systemic Inflammation with Pulmonary Infiltrates, Ventilator-Associated Pneumonia, Respiratory Distress, Intraventricular Hemorrhage, Abdominal Distention, Hypotension, Acidosis, Thrombocytopenia, Respiratory Failure, Purulent Secretions	
	*Elizabethkingia meningoseptica*	Spinal Muscular Atrophy, Prune Belly Syndrome, Hydrocephaly, Seizures, Down Syndrome, Necrotizing Enterocolitis, Pneumonia	Patients aged 4 to 11 months, ventilator water reservoir sample	2 Recovered, 4 Died	Not mentioned	
	*Elizabethkingia anophelis*	Intracranial Mass	6-Month-old infant	Death	Seizures, Tachypnea, Fever	
	*Elizabethkingia anophelis*	Congenital Tracheomalacia, Cerebral Palsy, Epilepsy	11-Year-old patient	Death	Desaturation, Fever	
	*Chryseobacterium and Elizabethkingia*	Malignancy, Bacteremia, Central Line–Associated Bloodstream Infection	Median age: 14 years; 29 patients with *Elizabethkingia*, 20 with *Chryseobacterium*	43 Recovered, 6 Died	Not mentioned	

The table summarizes documented cases of *Elizabethkingia* (and closely related genera) infections reported in various countries. Each row provides details on the causative agent, the disease or clinical condition, the infection source, the patient’s treatment outcome, the observed symptoms, and the relevant references. the data illustrate the diverse clinical presentations, affected populations, and infection origins associated with *Elizabethkingia* species in different healthcare and environmental contexts

### Comparative antibiotic profiles of Elizabethkingia in MENA regions

**Fig. 3:**
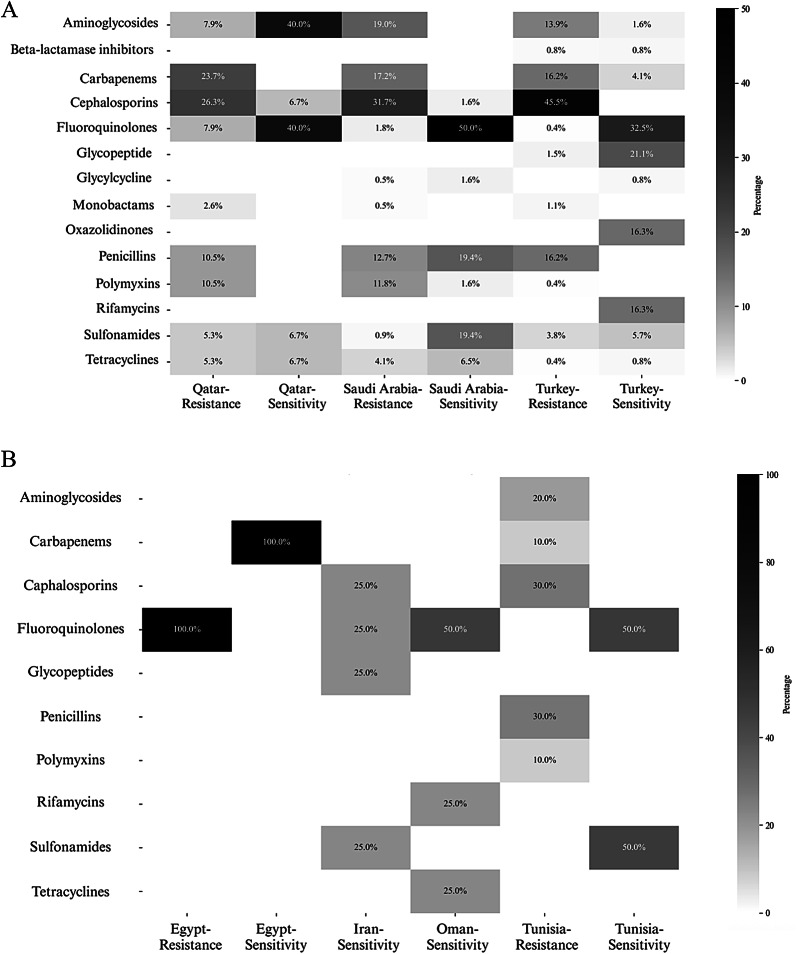
Comparative Heatmaps of Antibiotic Resistance and Sensitivity in *Elizabethkingia* across the MENA Region. Panel A shows data for Qatar, Saudi Arabia, and Turkey, while Panel B covers Egypt, Iran, Oman, and Tunisia. Each cell represents the percentage of isolates classified as resistant or sensitive to a given antibiotic family, with darker shades indicating higher percentages and white corresponding to zero. Antibiotic classes not displayed in a panel were not used or not reported in the respective studies. For each country, the left column shows the percentage of isolates classified as resistant, and the right column shows the percentage classified as sensitive. Cell color follows a single continuous gradient (white = 0%, darkest = 100%)

In Fig. 3A, which compares the resistance and sensitivity profiles per antibiotic families among *Elizabethkingia* strains isolated from Qatar, Saudi Arabia, and Turkey, the data indicate that fluoroquinolones antibiotics (mainly Ciprofloxacin and Levofloxacin) had the highest susceptibility rates among the tested isolates in these three countries. This finding suggests that fluoroquinolones may retain substantial efficacy against *Elizabethkingia*, aligning with their broad-spectrum activity and frequent clinical use. Beyond fluoroquinolones, other families display varied profiles across the three regions. In Qatar, for instance, cephalosporins and carbapenems show notable representation in resistant isolates, whereas aminoglycosides appear more often in sensitive categories. Similarly, Saudi Arabia with a broader range of antibiotic families with intermediate to high percentages in the resistant category—particularly carbapenems, cephalosporins, and polymyxins—reflecting a possible trend of multidrug resistance. However, certain families, such as sulfonamides, maintain lower yet meaningful proportions in the sensitive category after fluoroquinolones, hinting at the viability of alternative regimens. Turkey similarly exhibits high proportions of cephalosporins and carbapenems among resistant isolates, but also shows a strong susceptibility profile for fluoroquinolones, reinforcing their potential effectiveness in this setting.

These three countries (Qatar, Saudi Arabia, and Turkey) were grouped because they feature multiple reported resistance and sensitivity profiles in the literature, unlike other parts of the region that contributed only limited data. The observed differences in antibiotic family distributions may reflect local prescribing practices, historical usage patterns, and underlying resistance mechanisms of *Elizabethkingia* strains. In Fig. 3B, which displays antibiotic family distributions for *Elizabethkingia* strains reported from the remaining countries (e.g., Egypt, Iran, Oman, Tunisia, etc.), the overall profiles are more limited, often stemming from just one or two published isolates per region. Despite the smaller dataset, fluoroquinolones continue to appear as the most consistently effective option, aligning with the patterns observed in Qatar, Saudi Arabia, and Turkey. Certain families such as carbapenems or cephalosporins occasionally show high resistance rates, while others, including sulfonamides and glycopeptides, are sparsely represented and may require further study to confirm their clinical impact. Overall, the prominence of fluoroquinolones as a potentially effective option—together with the diverse, sometimes high, resistance rates to carbapenems and cephalosporins—indicate the importance of continuous surveillance and nuanced antibiotic stewardship when managing *Elizabethkingia* infections in these regions.

### Age distribution and treatment outcomes of Elizabethkingia infections

**Fig. 4:**
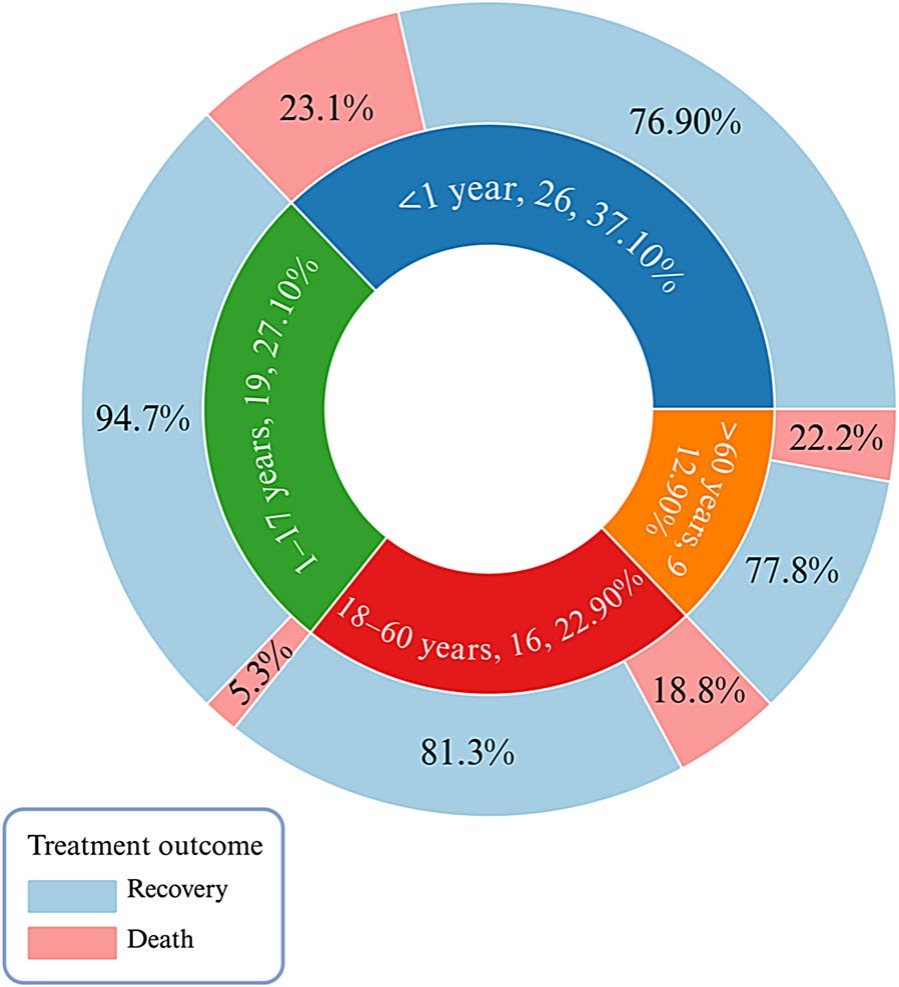
Distribution of *Elizabethkingia* Cases by Age Group and Treatment Outcome. Nested donut chart illustrating the distribution of *Elizabethkingia* cases by age group (inner ring) and corresponding treatment outcomes (outer ring). Each wedge in the inner ring represents a different age category, the number of reported cases, and the percentages. The outer ring subdivides these categories into Recovery (blue) and Death (red)

In Fig. 4, which depicts the distribution of *Elizabethkingia* cases by age group and associated treatment outcomes, a total of 70 cases were analyzed. Notably, 37.1% of all cases occurred in patients younger than one year, and this subgroup showed a 76.9% recovery rate versus a 23.1% death rate. The second largest category, children and adolescents aged 1–17 years (27.1% of total cases), had the most favorable outcomes, with 94.7% recovering and only 5.3% dying. Adults aged 18–60 years accounted for 22.9% of the cohort, exhibiting an 81.3% recovery rate and an 18.8% death rate. In contrast, individuals older than 60 years comprised 12.9% of the dataset, with recovery of 77.8% and 22.2% of death. Overall, 82.9% of the 70 cases recovered, while 17.1% succumbed to the infection.

These findings highlight two higher-risk populations at opposite ends of the age spectrum: neonates and infants (< 1 year) as well as the elderly (> 60 years). Although the youngest age group had the greatest absolute number of cases, they together with older adults exhibited relatively higher mortality compared to children aged 1–17 years. This observation aligns with the established clinical understanding that both neonatal and older adult patients may be more vulnerable to adverse outcomes, potentially due to immunologic immaturity in neonates and comorbidities in older adults. By contrast, the middle age groups (1–17 years and 18–60 years) generally fared better, especially in children and adolescents, where nearly all patients survived.

When considered alongside the antibiotic resistance profiles presented in Fig. 3A, B, these data collectively indicate that the choice of empiric therapy for *Elizabethkingia* should be informed by local resistance trends and by patient age. Notably, the majority of strains analyzed were tested primarily for fluoroquinolone susceptibility, reflecting their recognized efficacy and broad usage. Although this underscores the potential effectiveness of fluoroquinolones, further surveillance of alternative regimens across all age groups remains essential. In particular, neonates and older adults may require more aggressive or closely monitored treatment strategies. Meanwhile, the strong survival rate in children and adolescents highlights the potential benefit of timely, targeted antimicrobial regimens.

### Clinical spectrum of Elizabethkingia infections

Building on the antibiotic profiles described in the previous sections, the included studies reveal a diverse range of clinical diseases and symptoms caused by *Elizabethkingia* strains across the MENA region (Table 1). The most extensive data come from Turkey, followed by Saudi Arabia and Qatar, with sporadic reports from Egypt, Iran, Iraq, Jordan, Morocco, Oman, and Tunisia. Across these countries, *Elizabethkingia* infections span a wide clinical spectrum, from neonatal meningitis and sepsis to adult respiratory infections, biliary tract disease, and central line–associated bloodstream infections (CLABSIs). In Turkey, the majority of cases center on neonatal sepsis and meningitis, with several reports highlighting preterm infants suffering from respiratory failure, metabolic acidosis, and disseminated intravascular coagulation. Some pediatric cases involve underlying comorbidities such as thalassemia major, cystic fibrosis, or neurological disorders, whereas adults present with varied conditions, including pneumonia, postoperative cellulitis, and bacteremia.

In Saudi Arabia, *Elizabethkingia* and *Chryseobacterium* species were frequently associated with nosocomial infections across all age groups—including neonates, patients with chronic comorbidities (e.g., diabetes, cardiac disease, malignancy), and those requiring intensive care or prolonged hospitalization. Clinical presentations ranged from bacteremia and ventilator‑associated pneumonia to meningitis and sepsis, with features such as fever, hypotension, respiratory distress, and neurological deficits in neonates.

In Qatar, cases included neonatal meningitis, hemorrhagic colitis, and respiratory infections in older adults. Neonates typically presented with hypoactivity, irritability, bulging fontanelle, and fever, while adults showed signs of septic shock and severe respiratory failure, occasionally with co‑infections such as COVID‑19. Smaller case reports from Egypt, Iran, Iraq, Jordan, Morocco, Oman, and Tunisia described neonatal respiratory distress, convulsions, and sepsis, as well as diverse adult presentations ranging from biliary tract infections and ocular disease to hospital‑acquired pneumonia and bloodstream infections. Common clinical features across the region included fever, tachypnea, hypotension, and neurological deficits. Overall, these observations reinforce the importance of age-specific stewardship approaches and ongoing monitoring of *Elizabethkingia* resistance patterns to optimize clinical outcomes.

## Discussion

The analysis of the collected reports from different countries in the MENA region highlights a marked heterogeneity in the publication landscape of *Elizabethkingia* infections. Of 198 initially identified records, 35 articles met our stringent eligibility criteria, hinting at a temporal trend and geographic concentration that merits closer scrutiny. Turkey is the predominant contributor with 12 studies, followed by Saudi Arabia (9) and Qatar (4), while other countries such as Egypt, Iran, Iraq, Jordan, Tunisia, Morocco, and Oman provided limited data. The increase in publications from 2017 onwards—particularly in Saudi Arabia, Qatar, Iran, and later Tunisia—reflects a growing regional interest in understanding this pathogen, yet also exposes significant data gaps in several parts of the MENA region.

The analysis of the available AMR profiles demonstrates a notable predominance of fluoroquinolone susceptibility—particularly for ciprofloxacin and levofloxacin—among *Elizabethkingia* isolates in Qatar, Saudi Arabia, and Turkey as well as other MENA region countries (Fig. 3A, B). This observation aligns with earlier work in the literature including the research done by Lin et al. [43], who showed that although minocycline or tigecycline alone provided merely bacteriostatic activity against *Elizabethkingia anophelis*, co-administration of minocycline with either ciprofloxacin or levofloxacin considerably enhanced bacterial clearance in vitro and improved survival in a zebrafish infection model. The minocycline–levofloxacin combination yielded the greatest bacterial reduction, indicating a potentially synergistic avenue for employing fluoroquinolones alongside selective tetracycline-class agents to combat *Elizabethkingia* infections. Despite these positive indications for fluoroquinolones, resistance mechanisms are well documented. For instance, Jian et al., identified specific amino acid substitutions in GyrA—most frequently Ser83Ile or Ser83Arg, and less often Asp87Asn—as primary drivers of fluoroquinolone resistance, with no detected alterations in GyrB, ParC, or ParE [44]. The study also introduced a high-resolution melting assay capable of quickly differentiating wild-type *gyrA* from mutated variants, thereby enabling prompt identification of resistant isolates. While these *gyrA* substitutions largely explain the elevated MICs observed, the authors emphasize that efflux pumps, decreased permeability, and other mechanisms likely contribute to the overall fluoroquinolone resistance phenotype. Beyond clinical treatment considerations, Shaker et al. showed that *Elizabethkingia miricola*, in consortium with *Acinetobacter baumannii* and *Klebsiella pneumoniae*, can degrade ciprofloxacin and levofloxacin when these antibiotics serve as the sole carbon source [9]. Metabolic pathways—including hydrolysis, de-fluorination, and piperazine-ring cleavage—convert the compound into less active, more excretable forms via enzymes such as fluoroquinolone-acetylating aminoglycoside 6′-N-acetyltransferase and cytochrome P450 [9]. Such biodegradation highlights the potential environmental role of *Elizabethkingia* species in reducing antibiotic pollution—though it also raises questions about how microbial exposure could further drive resistance evolution. Another study by Lin et al. investigated the mutant prevention concentration (MPC) of ciprofloxacin and levofloxacin against *Elizabethkingia anophelis* and reported that most MPC values exceeded safe or achievable serum levels [45]. Maintaining fluoroquinolone concentrations above these thresholds would be impractical in clinical settings, suggesting that a careful management approach is recommended to prevent the emergence of resistant mutants.

The analysis of treatment outcomes stratified by age indicates significant variations in the clinical course of *Elizabethkingia* infections across the MENA region. Notably, neonates and infants (< 1 year) accounted for over one-third (37.1%) of the cases, exhibiting a recovery rate of 76.9% and a mortality rate of 23.1%. This high burden in the youngest patients can be attributed to the vulnerability of neonatal populations to severe infections, possibly due to their immunologic immaturity and underdeveloped host defenses. In contrast, the 1–17 years cohort, representing 27.1% of cases, showed better outcomes with a recovery rate of 94.7% and a lower mortality rate (5.3%). Adults aged 18–60 years and individuals older than 60 years, which comprised 22.9% and 12.9% of the cohort, respectively, demonstrated intermediate outcomes, with recovery rates of 81.3% and 77.8%, and mortality rates of 18.8% and 22.2% (Fig. 4).

When compared with the antibiotic resistance profiles presented in (Fig. 3A, B), these results emphasize that patient age must be a critical consideration in the selection of empiric therapy. The predominant testing for fluoroquinolone susceptibility across the studied strains—reflecting their broad clinical use and relatively favorable activity—supports the notion that fluoroquinolones, such as ciprofloxacin and levofloxacin, remain effective for many patients. However, the observed higher mortality rates among neonates and older adults, perhaps due to inefficient absorption, indicate that these high-risk groups might benefit from more aggressive or closely monitored therapeutic regimens. This is particularly pertinent given the dynamic resistance mechanisms inherent to *Elizabethkingia*, as evidenced by studies that have elucidated key target gene mutations in fluoroquinolone resistance [44].

From a broader clinical perspective, the diverse spectrum of *Elizabethkingia*-associated diseases in the MENA region—from neonatal sepsis and meningitis to adult-onset pneumonia amid complex co-morbidities—emphasizes the need for high clinical vigilance and strategic antimicrobial use. These findings highlight the importance of ongoing surveillance, age-tailored management strategies to reduce the morbidity and mortality linked to multidrug-resistant *Elizabethkingia* infections. Future research should focus on expanding surveillance to evaluate alternative antimicrobial regimens, rapid tools to detect resistance mechanisms, refining combination therapies, and elucidating the impact of environmental degradation and additional genetic or biochemical factors, such as efflux pumps, on fluoroquinolone efficacy. Although fluoroquinolones currently appear to be the most consistently effective agents against *Elizabethkingia*, the emergence of resistant subpopulations necessitates cautious interpretation and further study.

## Conclusions

This review highlights the growing clinical importance of *Elizabethkingia* infections within the MENA region, while identifying critical gaps that hinder a thorough understanding of disease characteristics and treatment effectiveness. Although the literature search encompassed publications from 2000 to 2024, only 35 studies met the inclusion criteria, of which nine lacked sufficient clinical details, limiting the depth of our analysis. The available evidence base is thus constrained by its limited size, methodological heterogeneity, and uneven geographical coverage, complicating efforts to draw definitive conclusions. Despite frequent reliance on fluoroquinolones in combination therapy regimens, the inconsistent and sparse treatment data available prevent a rigorous assessment of therapeutic effectiveness and its relationship to patient outcomes. To overcome these challenges, future studies should prioritize systematic, multicenter data collection, implement standardized clinical and treatment reporting protocols, and explore optimized therapeutic combinations. Addressing these areas is crucial for developing robust, evidence-based clinical guidelines and ultimately improving patient care in this emerging and clinically significant field.

## Electronic supplementary material

Supplementary Material 1

## Data Availability

The data presented in this study are available on request from the corresponding author.
